# Causal functional connectivity in Alzheimer's disease computed from time series fMRI data

**DOI:** 10.3389/fncom.2023.1251301

**Published:** 2023-12-19

**Authors:** Rahul Biswas, SuryaNarayana Sripada

**Affiliations:** ^1^Department of Electrical and Computer Engineering, University of Washington, Seattle, WA, United States; ^2^Center for Research on Science and Consciousness, Redmond, WA, United States

**Keywords:** causal inference, functional connectivity, brain mapping, directed graphical modeling, Alzheimer's disease, functional magnetic resonance imaging

## Abstract

*Functional connectivity* between brain regions is known to be altered in Alzheimer's disease and promises to be a biomarker for early diagnosis. Several approaches for functional connectivity obtain an un-directed network representing stochastic associations (correlations) between brain regions. However, association does not necessarily imply causation. In contrast, Causal Functional Connectivity (CFC) is more informative, providing a directed network representing causal relationships between brain regions. In this paper, we obtained the causal functional connectome for the whole brain from resting-state functional magnetic resonance imaging (rs-fMRI) recordings of subjects from three clinical groups: cognitively normal, mild cognitive impairment, and Alzheimer's disease. We applied the recently developed Time-aware PC (TPC) algorithm to infer the causal functional connectome for the whole brain. TPC supports model-free estimation of whole brain CFC based on directed graphical modeling in a time series setting. We compared the CFC outcome of TPC with that of other related approaches in the literature. Then, we used the CFC outcomes of TPC and performed an exploratory analysis of the difference in strengths of CFC edges between Alzheimer's and cognitively normal groups, based on edge-wise *p*-values obtained by Welch's *t*-test. The brain regions thus identified are found to be in agreement with literature on brain regions impacted by Alzheimer's disease, published by researchers from clinical/medical institutions.

## 1 Introduction

Alzheimer's disease (AD) is the most common age-related progressive neurodegenerative disorder. It typically begins with a preclinical phase and advances through mild cognitive impairment (MCI) to clinically significant AD, a form of dementia (Querfurth and LaFerla, 2010). Despite substantial efforts to identify biomarkers for AD, it still relies on clinical diagnosis, and early and accurate disease prediction remains limited (Laske et al., 2015; Li et al., 2019). Abnormal resting-state functional connectivity (FC) between brain regions has been observed as early as two decades before brain atrophy and the emergence of AD symptoms (Ashraf et al., 2015; Nakamura et al., 2017). Therefore, resting-state FC can potentially determine the relative risk of developing AD (Sheline and Raichle, 2013; Brier et al., 2014).

Resting-state functional magnetic resonance imaging (rs-fMRI) records the blood-oxygen-level-dependent (BOLD) signals from different brain regions while individuals are awake and not engaged in any specific task. The BOLD signal is popularly used to infer FC between brain regions partly due to the advantage that BOLD signal provides high spatial resolution (Yamasaki et al., 2012; Sporns, 2013; Liu et al., 2015; Xue et al., 2019).

FC refers to the stochastic relationship between brain regions with respect to their activity over time. Popularly, FC involves measuring the statistical association between signals from different brain regions. The statistical association measures are either pairwise associations between pairs of brain regions, such as Pearson's correlation, or multivariate i.e., incorporating multi-regional interactions such as undirected graphical models (Biswas and Shlizerman, 2022a). Detailed technical explanations of FC in fMRI can be found in Chen et al. (2017), Keilholz et al. (2017), and Scarapicchia et al. (2018). The findings from studies using FC (Wang et al., 2007; Kim et al., 2016), and meta-analyses (Jacobs et al., 2013; Li et al., 2015; Badhwar et al., 2017) indicate a decrease in connectivity in several brain regions with AD, such as the posterior cingulate cortex and hippocampus. These regions play a role in attentional processing and memory. On the other hand, some studies have found an increase in connectivity within brain regions in the early stages of AD and MCI (Gour et al., 2014; Bozzali et al., 2015; Hillary and Grafman, 2017). Such an increase in connectivity is a well known phenomenon that occurs when the communication between other brain regions is impaired. Such hyperconnectivity has been interpreted as a compensatory mechanism where alternative paths within the brain's network are recruited (Hillary and Grafman, 2017; Oldham and Fornito, 2019; Marek and Dosenbach, 2022).

In contrast to Associative FC (AFC), Causal FC (CFC) represents functional connectivity between brain regions more informatively by a directed graph, with nodes as the brain regions, directed edges between nodes indicating causal relationships between the brain regions, and weights of the directed edges quantifying the strength of the corresponding causal relationship (Spirtes et al., 2000). However, functional connectomics studies in general, and those concerning fMRI from AD in particular, have predominantly used associative measures of FC (Reid et al., 2019). There are a few studies that deal with comparing broad hypotheses of alteration within the CFC in AD (Rytsar et al., 2011; Khatri et al., 2021). However, this area is largely unexplored, partly due to the lack of methods that can infer CFC in a desirable manner, as explained next.

Several properties are desirable in the context of causal modeling of FC (Smith et al., 2011; Biswas and Shlizerman, 2022a). Specifically, the CFC should represent causality while free of limiting assumptions such as linearity of interactions. In addition, since the activity of brain regions are related over time, such temporal relationships should be incorporated in defining causal relationships in neural activity. The estimation of CFC should be computationally feasible for the whole brain FC instead of limiting it to a smaller brain network. It is also desirable to capture beyond-pairwise multi-regional cause-and-effect interactions between brain regions. Furthermore, since the BOLD signal occurs and is sampled at a temporal resolution that is far slower than the neuronal activity, thereby causal effects often appear as contemporaneous (Granger, 1969; Smith et al., 2011). Therefore, the causal model in fMRI data should support contemporaneous interactions between brain regions.

Among the methods for finding CFC, *Dynamic Causal Model* (DCM) requires a mechanistic biological model and compares different model hypotheses based on evidence from data, and is unsuitable for estimating the CFC of the whole brain (Friston et al., 2003; Smith et al., 2011). On the other hand, Granger Causality (GC) typically assumes a vector auto-regressive linear model for the activity of brain regions over time, and it tells whether a regions's past is predictive of another's future (Granger, 2001). Furthermore, GC does not include contemporaneous interactions. This is a drawback since fMRI data often consists of contemporaneous interactions (Smith et al., 2011). In contrast, *Directed Graphical Modeling* (DGM) has the advantage that it does not require the specification of a parametric equation of the neural activity over time, it is predictive of the consequence of interventions, and supports estimation of whole brain CFC. Furthermore, the approach inherently goes beyond pairwise interactions to include multi-regional interactions between brain regions and estimating the cause and effect of such interactions. The *Time-aware PC* (TPC) algorithm is a recent method for computing the CFC based on DGM in a time series setting (Biswas and Shlizerman, 2022b). In addition, TPC also accommodates contemporaneous interactions among brain regions. A detailed comparative analysis of approaches to find CFC is provided in Biswas and Shlizerman (2022a,b). With the development of methodologies such as TPC, it would be possible to infer the whole brain CFC with the aforementioned desirable properties.

In this paper, we apply the TPC algorithm to infer the CFC between brain regions from resting-state fMRI data. The TPC algorithm estimates the subject-specific CFC for each subject from their fMRI data. We compare the CFC outcome of TPC with GC and Sparse Partial Correlation (SPC), which are approaches to find the CFC and AFC, respectively. We then use the CFC outcome of TPC to investigate the alteration of CFC in AD. In this regard, we conducted an exploratory analysis for the difference in strength of causal connections in AD compared to CN subjects (and MCI compared to CN subjects), based on their edge-wise *p*-values given by Welch's *t*-test. We reported the resulting CFC edges with lowest edge-wise *p*-values for altered connectivity in AD compared to CN subjects and their corresponding brain regions. The brain regions identified in those analyses are consistent with published literature on regions impacted by AD, with each such publication being a report from a team involving a clinical setting and at least one medical expert, thereby validating the approach.

## 2 Materials and methods

### 2.1 Participants

The resting fMRI and demographic data were downloaded from the Alzheimer's Disease Neuroimaging Initiative (ADNI; http://adni.loni.usc.edu/). A total of 129 subjects were included in the study: 41 subjects who are CN, 54 subjects with MCI, and 34 subjects with AD.

Table 1 includes a summary of the participants' demographic and medical information. In the experiments, the subjects with AD presented significantly lower scores in the screening assessment cognitive test Mini-Mental State Examination (MMSE) in comparison with the other groups. The subjects were age-matched (Kruskal–Wallis test: *p* > 0.8), gender-matched (Chi-Squared test: *p* > 0.1), and matching number of years of education (Kruskal–Wallis test: *p* > 0.2). As expected, MMSE scores had a significant difference between all pairs of groups (Kruskal–Wallis test: *p* < 10^−14^).

**Table 1:** Summary of demographic information and Mini Mental State Examination (MMSE) for CN, MCI and AD subjects.

**Characteristic**	**CN**	**MCI**	**AD**	***p***
Number of subjects	41	54	34	–
Sex (M/F)	19/22	29/26	16/18	0.16
Age (years)	74.9 ± 6.4	74.2 ± 7.1	74.4 ± 7.4	0.86
Education (years)	16.5 ± 2.3	15.7 ± 2.6	15.4 ± 2.5	0.22
MMSE	29.1 ± 1.4	27.8 ± 1.9	21.9 ± 4.2	< 10^−14^

The second to fourth columns present group characteristics, mean ± SD. The fifth column presents *p*-values for the statistical significance of the inter-group differences. Differences in Sex was assessed using a Chi-Squared test and differences in Age, Education and MMSE using non-parametric analysis of variance by Kruskal–Wallis test.

### 2.2 Image acquisition

The acquisition of fMRI images was performed using Philips Medical Systems scanner. The fMRI images were obtained using an echo planar imaging sequence at a field strength of 3.0 Tesla, with a repetition time (TR) of 3 s, an echo time (TE) of 30 ms, and a flip angle of 80 degrees. The matrix size was 64 × 64 pixels, 140 volumes, 48 slices per volume, slice thickness of 3.3 mm, and voxel size of 3.3 × 3.3 × 3.3 mm^3^.

### 2.3 fMRI preprocessing

The fMRI pre-processing steps were carried out using the CONN toolbox version 21a, which utilizes the Statistical Parametric Mapping (SPM12), both of which are MATLAB-based cross-platform software (Friston et al., 1994; Nieto-Castanon and Whitfield-Gabrieli, 2021). We used the default pre-processing pipeline in CONN, consisting of the following steps in order: functional realignment and unwarp (subject motion estimation and correction), functional centering to (0, 0, 0) coordinates (translation), slice-time correction with interleaved slice order, outlier identification using Artifact Detection and Removal Tool, segmentation into gray matter, white matter and cerebrospinal fluid tissue, and direct normalization into standard Montreal Neurological Institute (MNI) brain space, and lastly, smoothing using spatial convolution with a Gaussian kernel of 8 mm full-width half maximum. This pipeline was followed by detrending and bandpass filtering (0.001–0.1 Hz) to remove low-frequency scanner drift and physiological noise in the fMRI images. The first four time points have been filtered out to remove any artifacts.

For the extraction of Regions-Of-Interest (ROIs), the automated anatomical labeling (AAL) atlas was utilized on the pre-processed rs-fMRI dataset (Tzourio-Mazoyer et al., 2002). The list of all regions in the AAL atlas is provided in Supplementary material along with their abbreviated, short, and full region names. This parcellation method has been demonstrated to be optimal for studying the FC between brain regions (Arslan et al., 2018). The voxels within each ROI were averaged, resulting in a time series for each ROI.

### 2.4 Inference of causal functional connectivity: Time-aware PC algorithm

**Figure 1:**
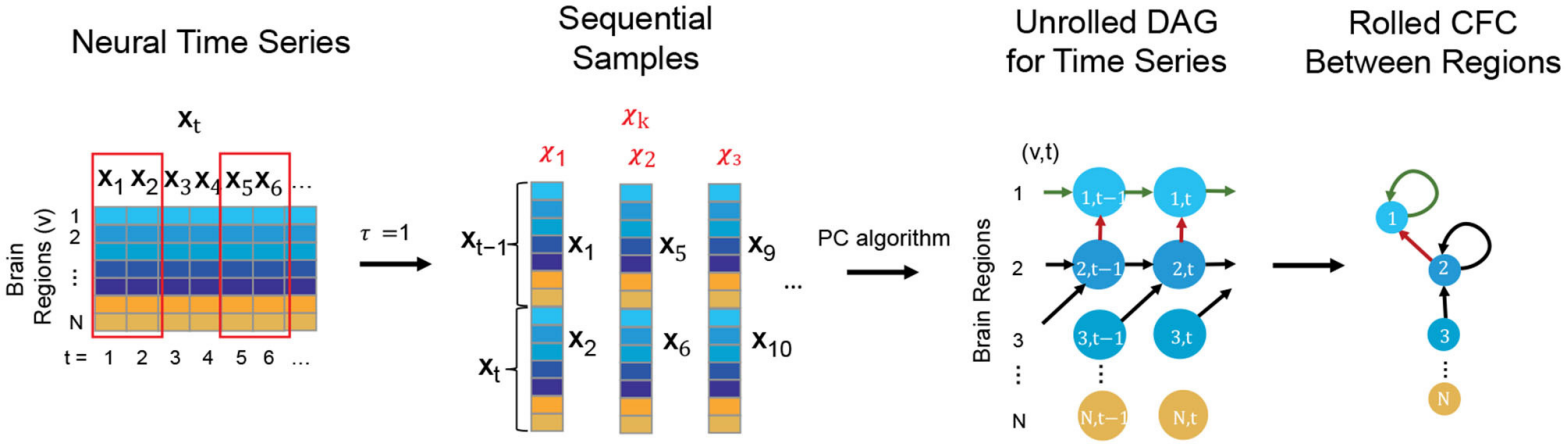
Steps conveying the concept of the TPC algorithm to infer CFC from observed neural time series data: First the neural time series is transformed to form sequential samples with a maximum time delay of interaction, τ (here τ = 1). Then, Peter-Clark (PC) algorithm is applied on the sequential samples to obtain the unrolled DAG satisfying the Directed Markov Property. Finally the unrolled DAG is transformed to obtain the Rolled CFC between regions.

The TPC Algorithm finds CFC between brain regions from time series based on DGM (Spirtes et al., 2000; Pearl, 2009; Biswas and Mukherjee, 2022; Biswas and Shlizerman, 2022a,b). While traditional DGM applies to static data, TPC extends the applicability of DGM to CFC inference in time series by first implementing the Directed Markov Property to model causal spatial and temporal interactions in the time series by an unrolled Directed Acyclic Graph (DAG) of the time series. The unrolled DAG consists of nodes (*v, t*), for region of interest *v* and time *t*, and edge (*v*_1_, *t*_1_) → (*v*_2_, *t*_2_) reflecting causal interaction from the BOLD signal in region *v*_1_ at time *t*_1_ to the BOLD signal in region *v*_2_ at time *t*_2_. The estimation of the unrolled DAG is carried out by first transforming the time series into sequential variables with a maximum time delay of interaction τ and then applying the Peter-Clark (PC) algorithm to infer the unrolled DAG based on the sequential variables (Kalisch and Bühlman, 2007). TPC then rolls the DAG back to obtain the CFC graph between the regions of interest (see Figure 1) (Biswas and Shlizerman, 2022b). We consider τ = 1 for our analyses, which would include interactions of the BOLD signal between regions of interest with a maximum time delay of 3 s, the TR of the fMRI acquisition. The Python package *TimeAwarePC* is used for implementation (Biswas and Shlizerman, 2022b).

The CFC outcome of this methodology is interpretable in the following manner: An edge from region *i*→*j* in the CFC estimate represents significant causal interaction from brain region *i* at preceding times to region *j* at following times. The model and the approach are non-parametric, meaning that it does not require the specification of a parametric dynamical equation for neural activity. The method captures beyond-pairwise multivariate interactions between brain regions. It also supports the estimation of the CFC for the whole brain in a computationally feasible manner. It also allows for time delays in interactions between the brain units and the presence of feedback loops. Furthermore, it has been shown that if the neural activity obeys an arbitrary dynamical process, the model outcome of TPC is consistent with respect to the causal relationships implied by the dynamical process and is predictive of counterfactual queries such as ablation or modulation (Biswas and Shlizerman, 2022b).

It is noteworthy that implementing the Directed Markov Property on the unrolled DAG to model causal relationships over time enables contemporaneous interactions e.g., from region *u* to region *v* at time *t* (Biswas and Shlizerman, 2022b). Such contemporaneous interactions are represented by the edge (*u, t*) → (*v, t*) in the unrolled DAG, and the presence of such an edge in the unrolled DAG would be reflected as an edge *u*→*v* in the Rolled CFC outcome. Such contemporaneous interactions are especially relevant in fMRI due to the relatively slow temporal resolution of the BOLD signal compared to the underlying neural activity (Smith et al., 2011).

### 2.5 Comparison with functional connectivity using other approaches

In Biswas and Shlizerman (2022b), the authors have demonstrated that TPC performs better in computing CFC compared to other methods such as GC on simulated and public benchmarking datasets as well as on a real neurobiological dataset of single neuron signals obtained using Neuropixels. Additionally, the authors have drawn contrast [in Biswas and Shlizerman (2022b)] with SPC, which is a popular method for inferring AFC. In this paper, we computed AFC using SPC and CFC using GC from fMRI data (Deshpande et al., 2009; Schouten et al., 2016). We compared these two outputs with the CFC obtained by TPC from fMRI data. The GC graph is computed using the Nitime Python library, which fits a Multi-variate Auto-Regressive (MVAR) model followed by the use of GrangerAnalyzer to compute the GC (Rokem et al., 2009). We consider MVAR model of order 1, and GC likelihood ratio statistic of greater than 95 percentile as indicating edges (Schmidt et al., 2016). The SPC was estimated by Graphical Lasso penalized Maximum Likelihood Estimation, whose optimal penalization was obtained by a five-fold cross-validation (Friedman et al., 2008).

### 2.6 Alterations of CFC edges in Alzheimer's disease

We perform an exploratory analysis of statistical trends for edge-wise inter-group differences. Using the subject-specific CFC computed by TPC algorithm, for each detected CFC edge, we reported the *p*-value in the Welch's *t*-test for greater average edge weight in one clinical group compared to another clinical group (Yuen, 1974). Specifically, we listed the CFC edges with 10 lowest *p*-values for greater average weight in CN compared to AD group (and for greater average weight in AD compared to CN). For a CFC edge from region *u* to region *v*, we refer to *u* as the *source* brain region and *v* as the *destination* brain region. The source brain regions of the CFC edges with lowest *p*-values are found to be in agreement with literature for regions impacted by AD.

## 3 Results

### 3.1 Subject-specific causal functional connectivity

**Figure 2:**
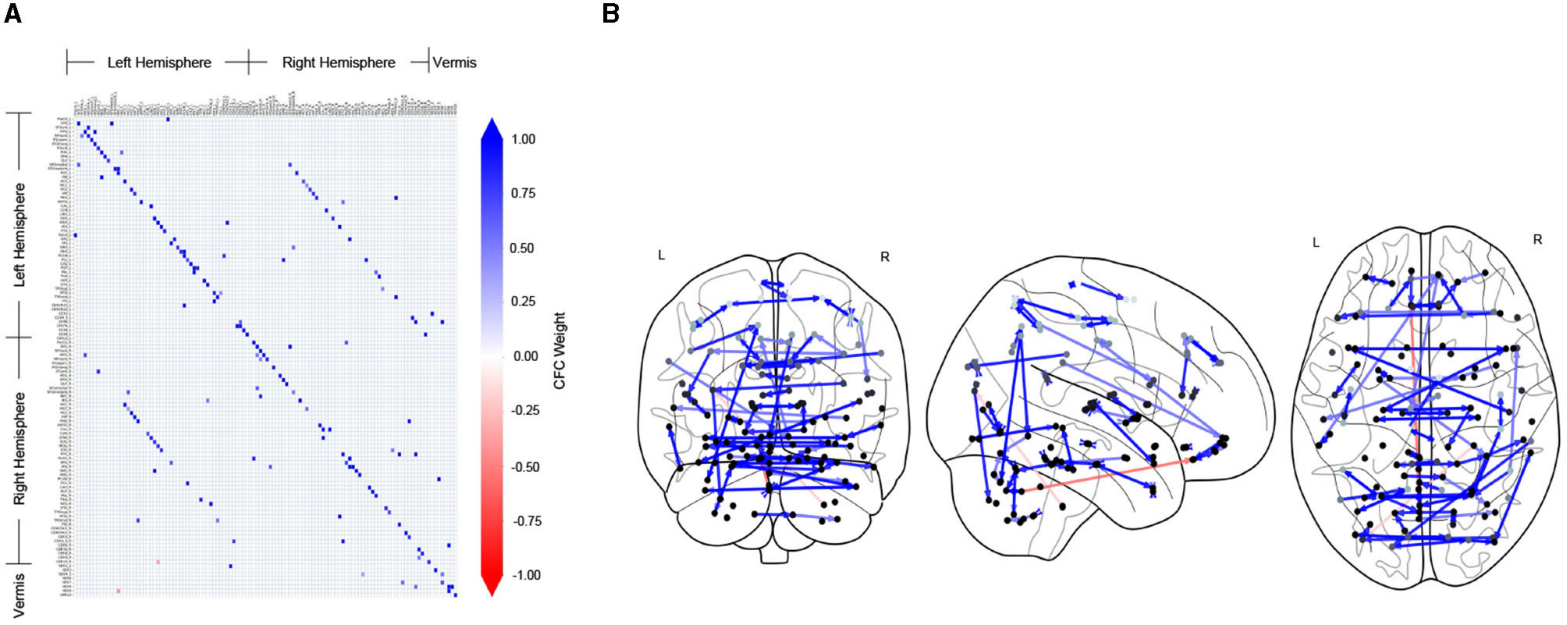
CFC for an example subject who is CN, estimated by TPC algorithm. **(A)** The estimated CFC is represented by its adjacency matrix, whose non-zero entry (*i, j*) represents the connection of region *i*→*j*. **(B)** The CFC is visualized with directed graph edges on the Frontal, Axial and Lateral brain maps (left to right). The nodes correspond to brain region centers, ranging from superficial (light gray) to deeper (darker gray) regions, in the AAL brain atlas.

Figure 2 shows the CFC estimated using the TPC algorithm for an example subject (ID: 129_S_4396) in the CN group. In Figure 2A, the CFC is represented in the form of a matrix, whose entry (*i, j*) indicates the presence of connectivity from region index *i*→*j*, and the value at entry (*i, j*) represents the weight of that causal connection. A positive value (blue) of the weight indicates excitatory influence, whereas a negative value (red) indicates inhibitory influence. The diagonal of the matrix representing self-connections for regions has been filtered out. In Figure 2B, the CFC is represented by a directed graph overlayed on schematics of the brain. The schematics of the brain comprise 2-dimensional brain projections in the Frontal, Axial, and Lateral planes. The nodes of the CFC graph correspond to the centers of brain regions in the AAL atlas. The nodes are colored light to dark gray according to their depth in the brain, with light gray representing superficial and dark gray representing deeper brain regions. The CFC graph provides a highly informative map of causal interactions between brain regions.

It is noteworthy that the CFC computed by TPC is sparse since the edges are filtered by conditional dependence tests. We quantified the sparsity of a CFC graph by its edge density. Edge density of a directed graph is the proportion of the number of edges in the directed graph over the total number of edges in the corresponding fully connected graph. Therefore, the edge density of an empty graph is 0 and that of a fully connected graph is 1. For the CFC graphs computed by TPC, the edge density for subjects in the CN group is (mean ± standard deviation) 0.0117 ± 0.0008, MCI group is 0.0118 ± 0.0009, and AD group is 0.0118 ± 0.0008, indicating a sparse CFC outcome of TPC for subjects in each of the groups.

### 3.2 Comparison with functional connectivity using other approaches

**Figure 3:**
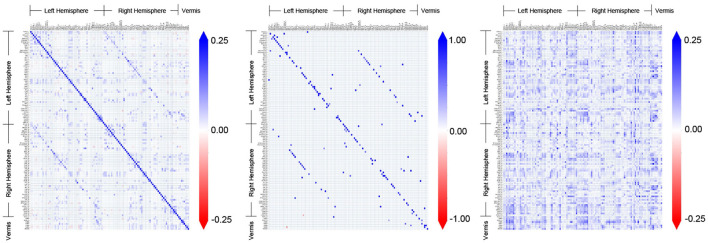
Comparison and demonstration of FC inferred by three methods: Associative FC using SPC, and Causal FC using TPC and GC. The estimated FC is represented by its adjacency matrix with edge weights, which is symmetric for Associative FC and asymmetric for Causal FC. In the adjacency matrices, a non-zero entry in (*i, j*) represents the connection of region *i*→*j*.

Figure 3 shows the adjacency matrices for the FC obtained by different methods for an example subject (ID: 129_S_4396) in the CN group. The AFC constitutes a distinct pattern of associative connectivity among the regions. It is expected that the CFC will be a directed subgraph of the AFC and be consistent with the overall patterns present in the AFC (Dadgostar et al., 2016; Wang et al., 2016). However, the patterns present in the CFC obtained by GC do not match with the AFC upon visual inspection. In comparison, the overall patterns present in the CFC obtained by TPC indeed match with the AFC obtained by SPC. On a detailed level, there are differences between TPC-CFC and AFC: TPC results in a directed graph thereby its adjacency matrix is asymmetric while AFC is an undirected graph with symmetric adjacency matrix. Furthermore, the CFC obtained by TPC includes self-loops represented by the diagonals of the adjacency matrix in contrast to GC, and results in a sparse matrix devoid of noise since the connections are filtered by conditional dependence tests.

### 3.3 Alterations of CFC edges in Alzheimer's disease

**Figure 4:**
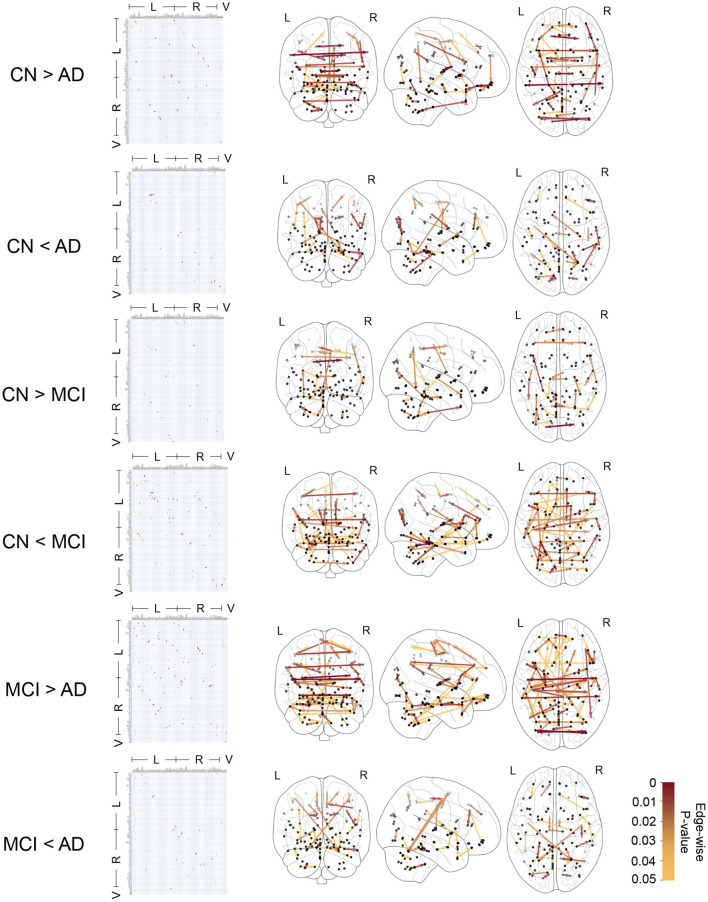
Causal functional connections with edge-weights differing between clinical groups with edge-wise *p*-values ranging in 0 − 0.05 based on *t*-test. The edge-wise *p*-values are represented by a matrix whose entry in (*i, j*) corresponds to the edge *i*→*j* and also represented by graph edges on brain schematics. The brain regions are annotated by Left (L) and Right (R) hemispheres of the brain and Vermis (V).

Figure 4 shows the edge-wise *p*-values for greater average edge weight in one clinical group compared to another, based on Welch's t-test. This provides insights into statistical trends for CFC edges that have an increase (or decrease) in strength in CN compared to MCI, CN compared to AD, and MCI compared to AD subjects.

In Table 2, we report 10 CFC edges that show the lowest *p*-values for greater average strength in CN subjects compared to AD, and their source brain regions. Similarly, we report another list of 10 CFC edges corresponding to greater average strength in subjects with AD compared to CN, and their source brain regions. The reported brain regions are in agreement with published medical literature cited in Table 2.

**Table 2:** CFC edges with lowest edge-wise *p*-values for (a) greater weight in CN compared to AD group and (b) greater weight in AD compared to CN group.

**Edge**	***p*-value**	**Region name**	**Reported by**
**(a) CN** > **AD**			
HES_L → ROL_L	0.0008	Heschl's gyrus	Hänggi et al., 2011; Dhanjal et al., 2013
ITG_R → ITG_R	0.001	Inferior temporal gyrus	Palmer and Burns, 1994; Scheff et al., 2011
SOG_L → SOG_R	0.001	Superior occipital gyrus	Beyer et al., 2009; Mao et al., 2021
SFG_L → SFG_R	0.001	Superior frontal gyrus	Brachova et al., 1993; Lue et al., 1996
MFGorb_R → IFGorb_R	0.002	Middle frontal gyrus	Neufang et al., 2011; Zhou et al., 2013
THA_R → THA_L	0.002	Thalamus	Braak and Braak, 1991; de Jong et al., 2008
SMG_L → SMG_R	0.002	SupraMarginal gyrus	Grignon et al., 1998; Desikan et al., 2009
IOG_L → IOG_R	0.005	Inferior occipital gyrus	Johnen et al., 2015; Wu et al., 2023
SMG_R → SMG_L	0.005	SupraMarginal gyrus	Grignon et al., 1998; Desikan et al., 2009
PCC_L → ANG_L	0.006	Posterior cingulate gyrus	Villain et al., 2008; Mascali et al., 2015; Caminiti et al., 2020
**(b) CN**<**AD**			
PHG_R → PHG_R	0.0008	Parahippocampal gyrus	Van Hoesen et al., 2000; Thangavel et al., 2008
REC_R → REC_R	0.004	Gyrus rectus	Mölsä et al., 1987; Nochlin et al., 1993; Sheline et al., 2010
CER6_R → CER4_5_R	0.008	Cerebellum	Joachim et al., 1989; Jacobs et al., 2018
IFGtriang_R → MFG_R	0.008	Inferior frontal gyrus	Eliasova et al., 2014; Cajanus et al., 2019
CER7b_L → ITG_R	0.008	Cerebellum	Joachim et al., 1989; Jacobs et al., 2018
FFG_R → ITG_R	0.008	Fusiform gyrus	Whitwell, 2010; Ma et al., 2020
CUN_L → SOG_L	0.014	Cuneus	He et al., 2007; Niskanen et al., 2011
VER1_2 → VER3	0.014	Vermis	Sjöbeck and Englund, 2001; A Mavroudis et al., 2013
CAL_L → SOG_L	0.014	Calcarine fissure	Ren et al., 2020; Yang et al., 2020
PCL_L → PCL_L	0.017	Paracentral lobule	Garcia Martin et al., 2013; Yang et al., 2019

The corresponding source brain regions are in agreement with regions reported in literature (right column) as impacted by AD.

## 4 Discussion

In this study, we have obtained the CFC of the whole brain from its resting state fMRI time series. We used the recently developed TPC algorithm based on directed graphical modeling in time series, to compute the CFC. In the dataset, the subjects belonged to three clinical categories: CN, MCI, and AD. We computed the subject-specific CFC using TPC and compared it with those obtained by other approaches, such as GC. We then used the CFC outcomes of TPC for further investigation into the alteration of CFC in AD. In this regard, we explored statistical trends for edges that have a difference in strength between clinical categories, based on their edge-wise *p*-values obtained by Welch's *t*-test. We reported the causal connections with lowest *p*-values for greater strength in CN compared to AD (and greater strength in AD compared to CN) and their corresponding brain regions. The brain regions identified in the above analyses were found to be in agreement with medical literature for regions impacted by AD.

In Figure 4 and Table 2, the presence of CFC edges with weight in AD greater than that in CN (in addition to edges with weight in AD less than that in CN) is consistent with published studies in the literature. While several studies have concluded decreased connectivity in MCI and AD compared to CN (Jacobs et al., 2013; Li et al., 2015; Badhwar et al., 2017), others have highlighted that MCI and early stages of AD can involve an increase in FC between brain regions (Fredericks et al., 2018; Penalba-Sánchez et al., 2023). This increase occurs when the communication between specific brain regions is impaired and has been interpreted as a compensatory mechanism where alternative paths within the brain's network are recruited (Hillary and Grafman, 2017; Oldham and Fornito, 2019; Marek and Dosenbach, 2022). In the short term, the augmentation of FC along alternative pathways exhibits efficiency and adaptability of the brain. However, it is imperative to acknowledge the susceptibility of these densely interconnected hubs to beta-amyloid deposition, which can elicit secondary damage through metabolic stress, ultimately culminating in system breakdown (Hillary and Grafman, 2017). Consequently, the initial state of hyperconnectivity observed in neurodegenerative disorders may gradually transition into hypoconnectivity among the engaged pathways, thereby contributing to cognitive decline as the disease advances (Marek and Dosenbach, 2022).

In Table 2A, the Heschl's gyrus (Heschl's gyrus Left → Rolandic operculum Left with edge-wise *p*-value 0.0008) is prominent for lower CFC weight in AD compared to CN subjects. The Heschl's gyrus is not only important for language comprehension, but it also has a crucial role in speech production, phonologic retrieval, and semantic processing (Warrier et al., 2009; Fernández et al., 2020), and has been reported in the literature to be impacted by AD (Hänggi et al., 2011; Dhanjal et al., 2013). The Thalamus is also present among the list of regions in Table 2A (Thalamus Right → Thalamus Left with edge-wise *p*-value 0.002). The Thalamus functions as a relay station between different sub-cortical areas and the cerebral cortex and also plays a role in sleep, wakefulness, consciousness, and memory (Steriade and Llinás, 1988; Gazzaniga et al., 2002; Aggleton et al., 2010; Bruno et al., 2013), and is also known to be impacted by AD (Braak and Braak, 1991; de Jong et al., 2008). Also present in the table is the Posterior cingulate gyrus (Posterior cingulate Left → Angular gyrus Left with edge-wise *p*-value of 0.006), which plays an essential role in memory integration and attentional processing, and is widely considered to be impacted by AD (Villain et al., 2008; Jacobs et al., 2013; Li et al., 2015; Badhwar et al., 2017). The Hippocampus, which is involved in long-term memory formation and memory retrieval, is not in the list of regions, yet exhibits a trend of reduction in CFC weight in AD compared to CN (Hippocampus Right → Parahippocampal gyrus Right, edge-wise *p*-value 0.033) (Boutet et al., 2014; Rao et al., 2022). Self-connections in Hippocampus have been reported to be often involved in compensatory mechanisms leading to increased strength in AD (Pasquini et al., 2015). In Table 2B, the self-connection Parahippocampal gyrus Right → Parahippocampal gyrus Right (edge-wise *p*-value 0.0008) is prominent for greater weight in AD compared to CN. It is known that the Parahippocampal gyrus is highly impacted by AD and is the focus of damage during disease onset, in a manner such that its connectivity to other regions of the brain decreases with AD, while its activity and intrinsic connectivity within the region increases with AD (Van Hoesen et al., 2000; Chen et al., 2014; Pasquini et al., 2015, 2016; Tahmasian et al., 2015).

TPC identified 1, 475 edges in the CFCs across subjects with CN and AD. To obtain the subset of edges which have significant inter-group difference at Bonferonni family-wise error rate of 0.05 requires a total of 6, 352 unique subjects across three groups (2, 117 per group) to ensure a family-wise power of 0.95 in detecting mean differences of a quarter of the standard deviation (Cohen's *D* = 0.25), computed by power_t_test function in MESS package in R (Ekstrøm, 2023). None of the databases that are available publicly have so many subjects. For example, ADNI has under 2,000 subjects (Weiner et al., 2017, https://adni.loni.usc.edu/adni-3/), and the Australian database has 2,359 subjects (Fowler et al., 2021, https://aibl.org.au/about/). Therefore we took a subset of the ADNI dataset that is captured using 3T fMRI scanner while matching education and age levels for exploratory analysis.

Based on the whole-brain CFC outcome alone, this study obtained brain regions that have been reported across more than 30 different studies of altered connectivity in AD, using different feature extraction methods and advanced imaging technologies (see Table 2). This demonstrates the promise of CFC computed by the TPC algorithm based on directed graphical models in a time series setting. Given the nature of AD, progressively more and more regions of the brain get impacted. Therefore, we make the case for the collection of larger datasets to enable the identification, at desirable levels of significance, of various subnetworks that alter with AD. This would promote the maturation and the use of the TPC-CFC (and other approaches) for prognostic and diagnostic purposes for AD.

It is noteworthy that machine-learning-based classifiers can help predict the clinical category of subjects and diagnose AD (Zhang et al., 2011, 2014; Gray et al., 2013; Salehi et al., 2020; Wen et al., 2020). Recently, researchers have proposed robust multi-class classification methods in the presence of incorrect labeling of classes using the broad learning system (Jin et al., 2021, 2023). Such classifiers would be able to classify a subject as belonging to one of the clinical categories, given a subject's fMRI time-series data as input. However, such classifiers do not compute the CFC between brain regions. Computing the CFC can nicely complement a classifier by providing insights into specific causal functional connections and subnetworks that are altered by AD (Chen et al., 2011; Du et al., 2018). Abnormal resting-state FC between brain regions is known to predate brain atrophy and the emergence of AD symptoms by upto two decades or more (Sheline and Raichle, 2013; Brier et al., 2014; Ashraf et al., 2015; Nakamura et al., 2017). Therefore, a subject's computed CFC can shed light on such abnormalities and promises to be a biomarker for early diagnosis and prognosis of the disease.

In this paper, we have demonstrated the following: (a) Application of the TPC algorithm to compute whole-brain CFC for each subject, (b) Comparison of CFCs computed using other approaches, (c) Interpretation of CFC in the context of AD using domain (neuropathological) knowledge, and (d) Exploratory analysis for edge-wise differences and corresponding brain regions with altered connectivity in subjects with AD compared to CN. The findings are consistent with published medical literature. In summary, our results show the promise of computing the whole-brain CFC from fMRI data using the TPC algorithm to gain prognostic and diagnostic insights.

## Data availability statement

Publicly available datasets were analyzed in this study. This data can be found at: http://adni.loni.usc.edu.

## Ethics statement

Ethical review and approval was not required for the study on human participants in accordance with the local legislation and institutional requirements. Written informed consent from the patients/ participants or patients/participants' legal guardian/next of kin was not required to participate in this study in accordance with the national legislation and the institutional requirements.

## Author contributions

RB and SS initiated the study, developed the methods, verified the results, and wrote and edited the manuscript. RB implemented the methods. All authors contributed to the article and approved the submitted version.
