# Supplementary material for: Causal functional connectivity in Alzheimer's disease computed from time series fMRI data
Source: Front Comput Neurosci. 2023 Dec 19;17:1251301. doi: 10.3389/fncom.2023.1251301 (PMC10758424; doi:10.3389/fncom.2023.1251301)
Supplement: Supplementary file 1 [file Table_1.pdf]

frontiers

Supplementary Material

1 AUTOMATED ANATOMICAL LABELING (AAL) ATLAS

The regions in the AAL atlas along with their abbreviated, short and full names are listed in Table S1.

Table S1: Names of regions in the AAL Atlas

No	Abbr. Name	Short Name	Full Region Name
1	PreCG_L	Precentral_L	Precentral gyrus Left
2	PreCG_R	Precentral_R	Precentral gyrus Right
3	SFG_L	Frontal_Sup_L	Superior frontal gyrus, dorsolateral Left
4	SFG_R	Frontal_Sup_R	Superior frontal gyrus, dorsolateral Right
5	SFGorb_L	Frontal_Sup_Orb_L	Superior frontal gyrus, pars orbitalis Left
6	SFGorb_R	Frontal_Sup_Orb_R	Superior frontal gyrus, pars orbitalis Right
7	MFG_L	Frontal_Mid_L	Middle frontal gyrus Left
8	MFG_R	Frontal_Mid_R	Middle frontal gyrus Right
9	MFGorb_L	Frontal_Mid_Orb_L	Middle frontal gyrus, pars orbitalis Left
10	MFGorb_R	Frontal_Mid_Orb_R	Middle frontal gyrus, pars orbitalis Right
11	IFGoperc_L	Frontal_Inf_Oper_L	Inferior frontal gyrus, opercular part Left
12	IFGoperc_R	Frontal_Inf_Oper_R	Inferior frontal gyrus, opercular part Right
13	IFGtriang_L	Frontal_Inf_Tri_L	Inferior frontal gyrus, triangular part Left
14	IFGtriang_R	Frontal_Inf_Tri_R	Inferior frontal gyrus, triangular part Right
15	IFGorb_L	Frontal_Inf_Orb_L	Inferior frontal gyrus, pars orbitalis, Left
16	IFGorb_R	Frontal_Inf_Orb_R	Inferior frontal gyrus, pars orbitalis, Right
17	ROL_L	Rolandic_Oper_L	Rolandic operculum Left
18	ROL_R	Rolandic_Oper_R	Rolandic operculum Right
19	SMA_L	Supp_Motor_Area_L	Supplementary motor area Left
20	SMA_R	Supp_Motor_Area_R	Supplementary motor area Right
21	OLF_L	Olfactory_L	Olfactory cortex Left
22	OLF_R	Olfactory_R	Olfactory cortex Right
23	SFGmedial_L	Frontal_Sup_Medial_L	Superior frontal gyrus, medial Left
24	SFGmedial_R	Frontal_Sup_Medial_R	Superior frontal gyrus, medial Right
25	SFGmedorb_L	Frontal_Med_Orb_L	Superior frontal gyrus, medial orbital Left
26	SFGmedorb_R	Frontal_Med_Orb_R	Superior frontal gyrus, medial orbital Right
27	REC_L	Rectus_L	Gyrus rectus Left
28	REC_R	Rectus_R	Gyrus rectus Right
29	INS_L	Insula_L	Insula Left
30	INS_R	Insula_R	Insula Right
31	ACC_L	Cingulum_Ant_L	Anterior cingulate & paracingulate gyri Left
32	ACC_R	Cingulum_Ant_R	Anterior cingulate & paracingulate gyri Right
33	MCC_L	Cingulum_Mid_L	Middle cingulate & paracingulate gyri Left
34	MCC_R	Cingulum_Mid_R	Middle cingulate & paracingulate gyri Right
35	PCC_L	Cingulum_Post_L	Posterior cingulate gyrus Left

36	PCC_R	Cingulum_Post_R	Posterior cingulate gyrus Right
37	HIP_L	Hippocampus_L	Hippocampus Left
38	HIP_R	Hippocampus_R	Hippocampus Right
39	PHG_L	ParaHippocampal_L	Parahippocampal gyrus Left
40	PHG_R	ParaHippocampal_R	Parahippocampal gyrus Right
41	AMYG_L	Amygdala_L	Amygdala Left
42	AMYG_R	Amygdala_R	Amygdala Right
43	CAL_L	Calcarine_L	Calcarine fissure and surrounding cortex Left
44	CAL_R	Calcarine_R	Calcarine fissure and surrounding cortex Right
45	CUN_L	Cuneus_L	Cuneus Left
46	CUN_R	Cuneus_R	Cuneus Right
47	LING_L	Lingual_L	Lingual gyrus Left
48	LING_R	Lingual_R	Lingual gyrus Right
49	SOG_L	Occipital_Sup_L	Superior occipital gyrus Left
50	SOG_R	Occipital_Sup_R	Superior occipital gyrus Right
51	MOG_L	Occipital_Mid_L	Middle occipital gyrus Left
52	MOG_R	Occipital_Mid_R	Middle occipital gyrus Right
53	IOG_L	Occipital_Inf_L	Inferior occipital gyrus Left
54	IOG_R	Occipital_Inf_R	Inferior occipital gyrus Right
55	FFG_L	Fusiform_L	Fusiform gyrus Left
56	FFG_R	Fusiform_R	Fusiform gyrus Right
57	PoCG_L	Postcentral_L	Postcentral gyrus Left
58	PoCG_R	Postcentral_R	Postcentral gyrus Right
59	SPG_L	Parietal_Sup_L	Superior parietal gyrus Left
60	SPG_R	Parietal_Sup_R	Superior parietal gyrus Right
61	IPG_L	Parietal_Inf_L	Inferior parietal gyrus, excluding supramargina...
62	IPG_R	Parietal_Inf_R	Inferior parietal gyrus, excluding supramargina...
63	SMG_L	SupraMarginal_L	SupraMarginal gyrus Left
64	SMG_R	SupraMarginal_R	SupraMarginal gyrus Right
65	ANG_L	Angular_L	Angular gyrus Left
66	ANG_R	Angular_R	Angular gyrus Right
67	PCUN_L	Precuneus_L	Precuneus Left
68	PCUN_R	Precuneus_R	Precuneus Right
69	PCL_L	Paracentral_Lobule_L	Paracentral lobule Left
70	PCL_R	Paracentral_Lobule_R	Paracentral lobule Right
71	CAU_L	Caudate_L	Caudate nucleus Left
72	CAU_R	Caudate_R	Caudate nucleus Right
73	PUT_L	Putamen_L	Lenticular nucleus, Putamen Left
74	PUT_R	Putamen_R	Lenticular nucleus, Putamen Right
75	PAL_L	Pallidum_L	Lenticular nucleus, Pallidum Left
76	PAL_R	Pallidum_R	Lenticular nucleus, Pallidum Right
77	THA_L	Thalamus_L	Thalamus Left
78	THA_R	Thalamus_R	Thalamus Right
79	HES_L	Heschl_L	Heschl's gyrus Left
80	HES_R	Heschl_R	Heschl's gyrus Right

81	STG_L	Temporal_Sup_L	Superior temporal gyrus Left
82	STG_R	Temporal_Sup_R	Superior temporal gyrus Right
83	TPOsup_L	Temporal_Pole_Sup_L	Temporal pole: superior temporal gyrus Left
84	TPOsup_R	Temporal_Pole_Sup_R	Temporal pole: superior temporal gyrus Right
85	MTG_L	Temporal_Mid_L	Middle temporal gyrus Left
86	MTG_R	Temporal_Mid_R	Middle temporal gyrus Right
87	TPOmid_L	Temporal_Pole_Mid_L	Temporal pole: middle temporal gyrus Left
88	TPOmid_R	Temporal_Pole_Mid_R	Temporal pole: middle temporal gyrus Right
89	ITG_L	Temporal_Inf_L	Inferior temporal gyrus Left
90	ITG_R	Temporal_Inf_R	Inferior temporal gyrus Right
91	CERCRU1_L	Cerebellum_Crus1_L	Crus I of cerebellar hemisphere Left
92	CERCRU1_R	Cerebellum_Crus1_R	Crus I of cerebellar hemisphere Right
93	CERCRU2_L	Cerebellum_Crus2_L	Crus II of cerebellar hemisphere Left
94	CERCRU2_R	Cerebellum_Crus2_R	Crus II of cerebellar hemisphere Right
95	CER3_L	Cerebellum_3_L	Lobule III of cerebellar hemisphere Left
96	CER3_R	Cerebellum_3_R	Lobule III of cerebellar hemisphere Right
97	CER4_5_L	Cerebellum_4_5_L	Lobule IV, V of cerebellar hemisphere Left
98	CER4_5_R	Cerebellum_4_5_R	Lobule IV, V of cerebellar hemisphere Right
99	CER6_L	Cerebellum_6_L	Lobule VI of cerebellar hemisphere Left
100	CER6_R	Cerebellum_6_R	Lobule VI of cerebellar hemisphere Right
101	CER7b_L	Cerebellum_7b_L	Lobule VIIB of cerebellar hemisphere Left
102	CER7b_R	Cerebellum_7b_R	Lobule VIIB of cerebellar hemisphere Right
103	CER8_L	Cerebellum_8_L	Lobule VIII of cerebellar hemisphere Left
104	CER8_R	Cerebellum_8_R	Lobule VIII of cerebellar hemisphere Right
105	CER9_L	Cerebellum_9_L	Lobule IX of cerebellar hemisphere Left
106	CER9_R	Cerebellum_9_R	Lobule IX of cerebellar hemisphere Right
107	CER10_L	Cerebellum_10_L	Lobule X of cerebellar hemisphere Left
108	CER10_R	Cerebellum_10_R	Lobule X of cerebellar hemisphere Right
109	VER1_2	Vermis_1_2	Lobule I, II of vermis
110	VER3	Vermis_3	Lobule III of vermis
111	VER4_5	Vermis_4_5	Lobule IV, V of vermis
112	VER6	Vermis_6	Lobule VI of vermis
113	VER7	Vermis_7	Lobule VII of vermis
114	VER8	Vermis_8	Lobule VIII of vermis
115	VER9	Vermis_9	Lobule IX of vermis
116	VER10	Vermis_10	Lobule X of vermis
